# 3D bioprinting for reconstituting the cancer microenvironment

**DOI:** 10.1038/s41698-020-0121-2

**Published:** 2020-07-27

**Authors:** Pallab Datta, Madhuri Dey, Zaman Ataie, Derya Unutmaz, Ibrahim T. Ozbolat

**Affiliations:** 1 Centre for Healthcare Science and Technology, Indian Institute of Engineering Science and Technology Shibpur, Howrah, India; 2 Department of Chemistry, Penn State University, University Park, PA USA; 3 Engineering Science and Mechanics Department, Penn State University, University Park, PA USA; 4 The Jackson Laboratory of Genomics Medicine, Farmington, CT USA; 5 The Huck Institutes of the Life Sciences, Penn State University, University Park, PA USA; 6 Biomedical Engineering Department, Penn State University, University Park, PA USA; 7 Materials Research Institute, Penn State University, University Park, PA USA

**Keywords:** Cancer microenvironment, Cancer microenvironment

## Abstract

The cancer microenvironment is known for its complexity, both in its content as well as its dynamic nature, which is difficult to study using two-dimensional (2D) cell culture models. Several advances in tissue engineering have allowed more physiologically relevant three-dimensional (3D) in vitro cancer models, such as spheroid cultures, biopolymer scaffolds, and cancer-on-a-chip devices. Although these models serve as powerful tools for dissecting the roles of various biochemical and biophysical cues in carcinoma initiation and progression, they lack the ability to control the organization of multiple cell types in a complex dynamic 3D architecture. By virtue of its ability to precisely define perfusable networks and position of various cell types in a high-throughput manner, 3D bioprinting has the potential to more closely recapitulate the cancer microenvironment, relative to current methods. In this review, we discuss the applications of 3D bioprinting in mimicking cancer microenvironment, their use in immunotherapy as prescreening tools, and overview of current bioprinted cancer models.

## Introduction

The escalating cost of drug development is a deterrent for conducting clinical trials, leading to a decrease in number of innovative treatments. For example, in oncology, the success rate of drugs entering clinical trials and obtaining Food and Drug Administration approval is only 5.1% (ref. ^1^). This situation offers an opportunity to stimulate the development of physiologically relevant tissue models with improved preclinical testing outcomes. Monolayer culture of cancer cells in two-dimensional (2D) environment is the simplest approach for in vitro cancer studies. Generally, 2D in vitro models represent only an oversimplified version of in vivo conditions and are not able to address many physiological questions. The tumor microenvironment is characterized by a bidirectional communication between the myriad cellular and noncellular components. Apart from biochemical signaling, various physical signaling like extracellular matrix (ECM) stiffness, topography, pattern, and interstitial flow, shear stresses, or fluid forces can influence the development of tumors. In a tumor lesion, an aberrant vascularization leads to oxygen, nutrient, and metabolic waste gradients causing the development of a necrotic core^2^. The cells in the core area adapt to a quiescent condition and are difficult to eradicate^3^. They also secrete hypoxia-inducible factors and other cytokines, which can alter the physiology of neighboring cells. Furthermore, cell–ECM interactions in the form of ECM remodeling, and recruitment of fibroblasts and perivascular and immune cells govern the metastasis behavior of malignant cells^4^. Thus, reconstruction of the complex microenvironment assumes great significance in modern cancer biology, which can be attempted in a three-dimensional (3D) model. Several approaches have been developed for 3D modeling of the tumor microenvironment, including spheroid culture, biopolymer scaffolds, and cancer-on-a-chip platforms. However, these models lack the capability to precisely control the location and organization of various cellular components in a tumor microenvironment.

### 3D bioprinting and its virtue in mimicking the tumor microenvironment

3D bioprinting is defined as the layer-by-layer deposition of bioinks, such as tissue spheroids, cell pellets, microcarriers, decellularized ECM (dECM) components, and cell-laden hydrogels, in a spatially defined manner as per a computer-aided designed structure to generate viable 3D constructs. In the last decade, bioprinting technologies have undergone remarkable advancements. Bioprinting modalities comprise of extrusion-based bioprinting (EBB), droplet-based bioprinting (DBB), and laser-based bioprinting (LBB), which have been described in details elsewhere^5,6^. EBB relies on robotic dispensing of continuous stream of bioinks under pneumatic- or motor-driven forces. The DBB modality, e.g., inkjet bioprinting, is based on deposition of droplets under thermal, piezoelectric, or solenoid-based mechanical actuation. LBB, generates constructs by deposition of bioinks in a pattern defined by a laser path. Bioprinting can be performed either in a scaffold-free or scaffold-based manner. In the scaffold-free approach, cell pellets or aggregates are bioprinted on a sacrificial material mold, such as alginate or agarose, which is discarded once the bioprinted tissue matures and subsequently deposits its own ECM components. In the scaffold-based approach, a 3D construct is printed using a bioink that comprises of cells encapsulated in a hydrogel. Scaffold-based bioprinting then relies on the degradation kinetics, as well as cell–material interactions to direct tissue growth^7–12^. We highlight the major advantages that bioprinting hold over other biofabrication techniques in the ensuing section.

### Advantages of bioprinting in reconstitution of the tumor microenvironment

#### Spatial control on matrix properties

ECM mechanics, such as matrix stiffness, plays a major role in the metastatic behavior of cancer cells and this factor can be incorporated in a 3D bioprinted tumor model^13^. 3D constructs of polyethylene glycol (PEG)-based log-pile micro architecture was formed with variable stiffness (0.9–5.5 MPa) for cell migration study^14^. 3D honeycomb structures resembling rat capillaries were also formed using the same method by the same group^15^. The effect of matrix stiffness and vessels diameter on cellular migration was tested, and the study showed that the migration speed of normal cells remained unaffected with altered vessel diameter, whereas the speed of HeLa cell migration considerably decreased with increasing vessel diameter^16^. In bioprinted cancer models, a gradient of hydrogel matrix was also developed in 3D to facilitate directional cell migration via controlled deposition of bioink mimicking the physiochemical environment of cancer cells^17^.

In addition to stiffness, spatial distribution of biochemical factors can also be mediated to mimic the native tumor microenvironment. Freeform reversible embedding of suspended hydrogels (FRESH) is one of the recent techniques of EBB methods for mimicking the complex native architecture of biological tissues^18,19^. Employing the FRESH technique, a neuroblastoma model was developed with sodium alginate^20^. Biomolecular gradient was facilitated through the release of 3D printed stimuli-responsive capsules. Such capsules consisted of an aqueous core of biomolecules coated with a poly(lactic-co-glycolic) acid-based polymer shell^17^. The capsules were loaded with plasmonic gold nanorods in the shell, which enabled selective rupture and release of biomolecules by controlling the wavelength of irradiation. Thus, 3D bioprinting facilitated precise control over location of these capsules in a hydrogel, which makes it a suitable carrier of biomolecular payloads. However, to obtain spatial control of matrix properties, it is essential to employ the scaffold-based bioprinting approach necessitating optimization of material properties, such as hydrogel density as well as biocompatibility, cell–material interactions, and chances of toxicity induced by scaffold degradation products. Moreover, different bioprinting approaches need to be developed involving multimaterial bioprinting, or gradient-based material deposition to study cancer metastasis across soft to hard tissue interfaces, such as breast cancer metastasis to bone, lungs, or brain.

#### Ability to integrate perfusable vascular networks

Bioprinting has been leveraged for fabrication of both blood vessels as well as vascularized tissues. This can be accomplished by either a scaffold-based or scaffold-free approach. In scaffold-based approach, the cell–biomaterial interactions with hydrogels assume significance and limit the number of cells in constructs, whereas scaffold-free method relies on self-assembly and fusion of cell aggregates. In the literature, all the three bioprinting modalities have been extensively explored for fabrication of vascular networks^21^. Often, bioprinting with a sacrificial bioink, which is dissolved subsequently to leave behind a porous network, have found utility as stand-alone bioprinting of overhang structures, which are usually difficult to fabricate. In this context, bioprinting based on a support bath have also attracted considerable attention^21^. The support bath provides a semisolid medium onto which water-rich, low viscosity bioinks can be bioprinted, overcoming the limitations of printing over flat air interfaces. Bioprinting in suspension bath is particularly attractive to fabricate structures, which can self-support themselves^22^. Perfusable vascular structures were incorporated in 3D architectures for studying the invasion of cancer cells through vessels. For recapitulating the initiation of cancer spreading, several chemometric pathways that guide cellular invasion and angiogenesis, can be programmed. For reconstructing the tumor microenvironment, individual 3D bioprinted components can be assembled from droplets of tumor cells obtained from primary tumor site, microchannels comprising endothelial cells, fibroblast containing hydrogel acting as tumor stroma, and programmable capsules for releasing chemical signals^23^. The effect of micro-scale architecture and mechanical fluid microenvironment on cancer cell phenotype, molecular signaling, cell cycle, gene expression, and functionality has been studied^24^. It was demonstrated that G2/M cell cycle arrest occurs under shear stress of the order of 12 dynes/cm^2^, whereas static culture conditions cause G0/G1 arrest. Decreased expressions of certain cyclins, and cyclin-dependent protein kinases mediated by αvβ3 and β1 integrins through Smad1 and Smad5 was observed as the probable pathway, thus providing a basis for relating mechanical cues with cell functions.

#### High-throughput fabrication of cancer models

Automation and high-throughput assay capabilities for metabolism, and toxicology are important processes for drug discovery and development. Presently, high-throughput screening (HTS) is performed with 2D cell cultures in multi-well plates. Hundreds to thousands of lead compounds can be tested in 96-well arrays, using small volumes and weights of candidate compounds by fast, mechanically controlled processes. However, quantification techniques in HTS, such as absorbance, or fluorescence measurements, need extensive standardization. HTS assays have been tremendously improved due to improvements in molecular biology and genomics, yielding phenotypically and genotypically well-characterized disease models and bioreactors with scalable culture characteristics. As the advantages of 3D culture over 2D cultures become more apparent and the role of heterogeneous tumor microenvironment comprising multiple cell types and cell–material interactions assume significance, bioprinting is emerging as a potential method for HTS assays, which can provide rapid process flows with higher consistency and increased sample volumes over manual deposition. Therefore, the bioprinting community also becomes sensitive to the needs for HTS, and improvements in process flow times, resolution, bioprinting speeds, as well as quantification techniques are pursued^25^. Amongst different bioprinting modalities, DBB is particularly suited for HTS fabrication. The modality can eject the bioinks from print heads in a highly synchronized manner at rates of ∼1000 droplets per second, to deposit 50–100 μm droplets maintaining high cell viability^26^. In contrast, LBB can achieve throughput rates of up to 20 Hz. Though, some DBB processes, such as inkjet bioprinting, can achieve higher printing speeds, droplet instabilities has been observed at high frequencies. Despite this fact, DBB has substantial potential to generate tumor tissue models in a high-throughput manner for use in HTS. Using various bioprinting modalities, in vitro models of standard 384- and 1536-well plate sizes^27^ have been reported. With respect to bioprinting to obtain HTS models, it is also necessary to standardize the assay end points, which will correlate with patient outcomes. Compared to 2D models, assays in 3D models is more challenging since the quantification of whole-construct florescence or spectroscopy readouts do not provide information at cellular-level resolution, critical for co-culture and investigations of heterotypic cell–cell interactions^25^.

## 3D bioprinted cancer models

In the forthcoming section, the progress made in in vitro models of different types of cancer fabricated by bioprinting are described in details. A summary of these achievements for each cancer model indicating the cell types, bioink, or substrate and the bioprinting modality used along with their major advantages/limitations are presented in Table 1.

**Table 1:** 3D bioprinted cancer models.

Cancer model type	Cell types used	Bioink or substrate used	Bioprinting modalities used	Ref.
Glioblastoma-on-a-chip	Glioma cell line U118 and endothelial cells	Collagen or dECM hydrogel	EBB	^28^
	Glioma stem cell (shell); glioma cell line (core)	Alginate	Coaxial EBB	^29^
	Glioma stem cells	Gelatin, alginate, and fibrinogen	EBB	^30^
	Hepatoma HepG2 and glioma cell U251	Alginate	DBB (Inkjet)	^31^
	iPSC-derived human neural progenitor cells and U118 human glioma cells	Scaffold-free 3D culture	EBB	^32^
	Human glioma stem cell line, U118	Sodium alginate and gelatin	EBB	^33^
	Glioblastoma-associated macrophages (GAMs) and glioblastoma cells	GelMA	EBB	^34^
	Human primary umbilical cord-derived mesenchymal stromal cells (UC-MSC, referred to as MSCs), HUVEC, and human bone marrow-derived epithelial-neuroblastoma immortalized cells (SH-SY5Y)	Agarose and type-I collagen	DBB	^100^
Breast tumor model	Immortalized non-tumorigenic human breast epithelial cell lines MCF-12A and MCF10A	Rat tail collagen I	EBB	^36^
	MCF-7 BC cells	PBS solution	DBB	^101^
	Immortalized non-tumorigenic human breast epithelial cell line, MCF-12A, and the breast carcinoma cell lines MCF-7 and MDA-MB-468	Rat tail collagen	EBB	^37^
	MCF-7 cell	Gelatin-PEG	DBB	^38^
	BT474 breast cancer cells, human perinatal foreskin fibroblasts (BJ), and human adult dermal fibroblasts (HDF)	Poly(ethylene glycol) diacrylate (PEGDA)	LBB (optical projection based)	^102^
	Primary breast cancer cells (21PT) and ADMSC	Methacrylated hyaluronic acid and gelatin	EBB	^40^
	Breast epithelial cell lines MCF10A, MCF10A-NeuN, MDA-MB-231, and MCF-7	Matrigel and gelatin-alginate	Coaxial EBB	^41^
	Mouse fibroblast (L929)	Alginate	EBB	^42^
	Breast cancer cell lines of distinct subtypes, luminal (MCF-7), basal like (HCC1143), HER2 amplified (SKBR3), and claudin low (MDA-MB-231)	Alginate and gelatin	EBB	^43^
	IMR-90 fibroblast cells and MDA-MB-231 cancer cells	Alginate and gelatin	EBB	^44^
	Primary human bone marrow MSCs, BrCa cell line	GelMA and nHA	LBB	^45^
	MDA-MB-231; human bone marrow stromal cells	Modified nHA	EBB	^104^
	Metastatic breast cancer cell line MDA-MB-231 Human fetal osteoblast cell line hFOB	PEG hydrogel and nHA	LBB (streolitography)	^103^
	MDA-MB-231 cells	PEG, PEGDA, and nHA	LBB (streolitography)	^47^
Pancreatic adenocarcinoma	Pancreatic cancer and stellate cells, endothelial cells	Alginate and gelatin	EBB	^48^
Ovarian cancer	Ovarian cancer OVCAR-5 and (MRC-5) fibroblasts	Matrigel	DBB	^49,50^
	Human ovarian cancer cell line (SKOV3) and human foreskin-derived fibroblasts (HFF)	GelMA	LBB (streolitography)	^52^
Cervical tumor	Hela cells	Gelatin, alginate, and fibrinogen	EBB	^51^
Hepatocarcinoma model	Human perinatal foreskin fibroblasts and human adult dermal fibroblasts	Liver dECM	LBB	^55^
	C3H/10T1/2, clone 8 cells, and GFP-expressing human neonatal dermal fibroblast cells; HUVECs and RFP-HUVECs	GelMA	EBB	^56^

### Glioblastoma

**Fig. 1:**
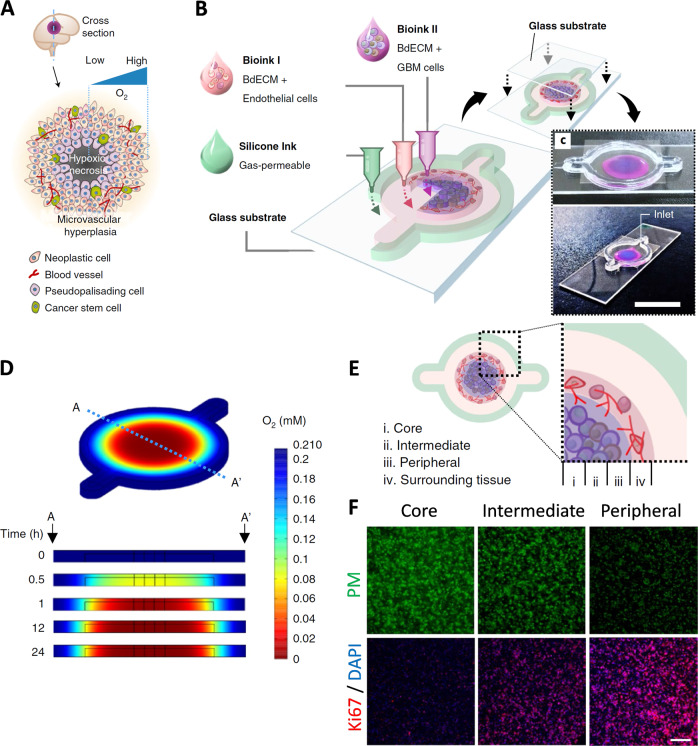
GBM-on-a-chip. **a** Schematic representation of a tumor cross-section depicting the hypoxic core and different biological components typically found in a tumor microenvironment. **b** Schematic illustration of the bioinks used to fabricate a compartmentalized GBM-on-a-chip model. **c** Mock representation of bioink compartments of brain dECM laden with HUVECs (depicted in magenta), and brain dECM with GBM cells (blue) shown from above (top) and from the corner (bottom; scale bar, 2 cm). **d** Computer simulation of oxygen gradient along A–A′ cross-section depicted by time-lapse jet colourmap images. **e** Schematic representation of the various regions within the printed GBM model (i) core, (ii) intermediate, (iii) peripheral regions, and (iv) the surrounding tissue region. **f** Fluorescent images of cross-section of immunostained tumor highlighting the hypoxic cells using pimonidazole (PM), Ki67 for the proliferating cells, and DAPI for the cell nuclei (scale bar, 200 μm.) (Reproduced/adapted with permission from ref. ^28^).

Glioblastoma (GBM) is the most common aggressive form of cancer affecting the central nervous system. It forms from star-shaped cells called astrocytes, present in the brain and spinal cord. GBM tumors are usually surgically removed followed by radiotherapy, chemotherapy, and other comprehensive treatment methods. However, patients often suffer from relapse of tumors caused by the drug resistance of glioma cells. Thus, it is essential to investigate the drug response of glioma cells using a suitable in vitro model. In this regard, a representative 3D bioprinted model, ‘glioblastoma-on-a-chip’, was established using patient-derived glioblastoma cells co-cultured with endothelial cells on dECM environment. In this model, a cancer-stroma concentric-ring structure was developed, which maintained the radial oxygen gradient and mimicked the in vivo tumor-like microenvironment (Fig. 1). The model showed clinical significance by reproducing patient-specific resistance to concurrent chemoradiation and temozolomide drug. Such a model may serve as the purpose of screening of effective treatment modalities for glioblastoma patients, who are resistant to conventional treatments^28^. In several studies, drug resistance models were extrusion bioprinted, employing human glioma stem cells encapsulated in alginate or gelatin-based hydrogels^29,30^. Higher drug resistance was observed for the 3D models as compared to 2D. Furthermore, inkjet bioprinting was also used to print a co-patterned hepatoma and glioma cell-based model to observe the anticancer activity of the drug tegafur on the glioma cells^31^. Apart from chemoresistance, tumor cell invasion into surrounding parenchymal tissue, post-surgery, is another driving factor for low response rates in glioma. This phenomenon was studied using 3D bioprinting combined with a scaffold-free culture technique. 3D confocal microscopy was used to observe glioma cell invasion into neural-like tissue spheroid derived from rodent neural progenitor cells^32^. Bioprinted tumor models were also developed to enhance the stemness properties of glioma cells, as glioma stem cells are an important mediator of cancer resistance. The model elucidated epithelial–mesenchymal transition and possible mechanism of glioma stem cells enhancement. A marked difference was evident in the drug resistance and in vivo tumorogenicity of cells when compared to 2D (ref. ^33^). Further, to study cellular interaction, a 3D bioprinted mini-brain model was developed by Heinrich et al. incorporating glioblastoma-associated macrophages (GAMs) and glioblastoma cells in glioblastoma multiforme. In this model, macrophages were actively recruited by glioblastoma cells, and metamorphosed into a typical GAM phenotype and upregulation of several genes, which was found to compare well with the transcriptomic analysis of over 150 patients with GBM. The macrophages were shown to be inducing the proliferation and invasion of glioblastoma cells. The bioprinted tumor model was also applied to study the inhibitory effect of drugs on GAMs and tumor cells^34^.

### Breast cancer

**Fig. 2:**
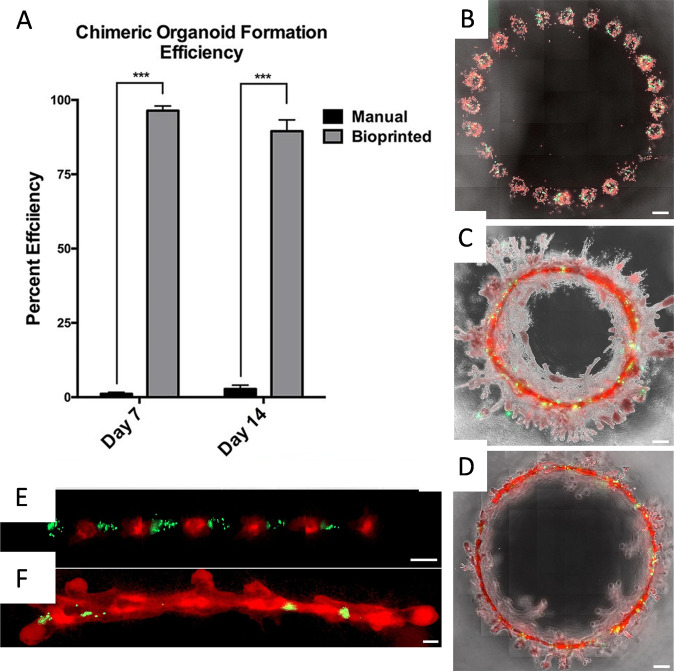
Chimeric organoids fabricated using bioprinting. **a** Formation of chimeric organoids was significantly better when 3D bioprinted compared to standard culture methods. ^***^p < 0.001 by two-way ANOVA. **b**–**d** Large chimeric organoids bioprinted in a circular pattern with 500 µm space between them, consisting of 5:1 ratio of MCF-12A (red) and MDA-MB-468 (green) cells at day 3 (**b**), day 7 (**c**), and day 21 (**d**). **e**, **f** A 300 µm spaced alternating prints of tumorigenic MDA-MB-468 cells (green) and MCF-12A cells (red) at day 1 (**e**) and day 7 (**f**) demonstrating incorporation of cancer cells into the organoid (scale bars: **b**–**d** = 500 µm; **e** and **f** = 200 µm; reproduced/adapted with permission from ref. ^37^).

Breast cancer is one of the most leading type of invasive cancer. This is a heterogenous cancer type and is aggravated due to the presence of a small population of stem cells that cause resistance to chemotherapy or radiation treatment, and result in recurrence. While considerable progress has been made in treatment of localized tumors, severe bottlenecks need to be overcome to remedy metastatic or recurrent cases. Importantly, breast cancer niche is characterized by the presence of surrounding ECM, endothelial and immune cells, fibroblasts, and adipocytes, which have established role in tumor progression^35^. Thus, there exists a constant need to study the breast tumor microenvironment and as such has also been studied using 3D bioprinting techniques. As organoid culture is one of the most promising techniques for studying cancer progression, a study was conducted by Reid et al. to fabricate breast tumor organoids employing bioprinting. In this study, a small number (as few as ten cells) of breast cancer cells was bioprinted in a single print and individual organoids were printed 500 μm apart. Bioprinted cells further fused and formed single layer of mammary cells and developed ~3 mm contiguous lumen circles^36^. On comparing a 2D and 3D bioprinted breast cancer model, bioprinted MCF-7 cells produced increased levels of chaperone proteins (like, HSP70 and HSP90), in the presence of high concentration of tamoxifen, thus closely mimicking native tumors. In a 3D bioprinted collagen model, biomimetic chimeric organoids were developed by co-bioprinting breast cancer cells and normal mammary epithelial cells. Comparing both structures (depicted in Fig. 2), the study showed the significant increase of 5-hydroxymethylcytosine in chimeric structure than the tumoroid one^37^. In other studies, breast cancer spheroids were formed on PEG-diacrylate concave structures through LBB (refs. ^38,39^). In long-term culture conditions, spheroids showed hypoxic core with the presence of necrotic cells. The close resemblance with pathological conditions enables the use of such a model as an efficient drug screening tool. Thus, 3D tumor spheroid models hold prospects to study microenvironmental control over tumorogenesis of breast cancer cells.

Scaffold-based 3D cancer models have also been widely used to study the effect of anticancer drugs. In this aspect, a 3D bioprinted breast cancer model was fabricated using primary breast cancer cells (21PT) and adipose-derived stem cells (ADSCs) and cellular responses were observed under the treatment of doxorubicin (DOX). Such a model allowed the establishment of a direct relationship between the thickness of ADSCs and their resistance to DOX (ref. ^40^). In another study, bioprinted spheroids were found to be more resistant to paclitaxel compared to the individually bioprinted cells^41^.

**Fig. 3:**
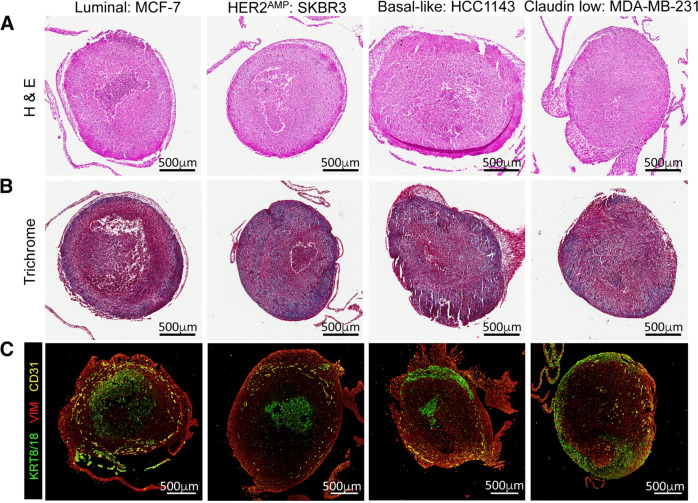
Breast cancer cells were extrusion bioprinted into a stromal mix of primary human mammary fibroblast and human umbilical vein endothelial cells (HUVECs). **a** Representative H&E images of bioprinted tissues, fixed on day 10. **b** Trichrome staining of bioprinted tissues. Scale bars, 500 µm. **d** Immunofluorescence images of bioprinted tissue sections, stained for KRT8/18 (green), VIM (red), and CD31 (yellow; reproduced/adapted with permission from ref. ^43^).

In most 3D cancer models, absence of stroma and immune cells fails to mimic the tumor microenvironment. A recent development sequentially incorporated both cancer and stromal cells in 3D hydrogels, where the volumetric viability of cells was determined through fluoro-D-oxy glucose staining and positron emission tomography–computerized tomography after three days of culture^42^. In another study, Langer et al. introduced a 3D bioprinted tumor model consisting of multiple cell types, including fibroblasts, adipocytes, patient-derived cancer cells, and endothelial cells, to examine the interaction between cancer and stromal cells (Fig. 3)^43^. This heterocellular tumor model recapitulated different aspects of neoplastic tissues and also allowed the investigations of cellular responses by manipulating tumor microenvironment under therapeutic interventions. Similarly, using a alginate/gelatin-based 3D bioprinted breast cancer model containing fibroblasts and MDA-MB-231 cancer cells, separated through a partition, showed spheroid formation of cancer cells and migration of fibroblasts to those spheroids^44^.

The normal mammary microenvironment can suppress tumorogenesis and redirect cancer cells to adopt a normal mammary epithelial cell fate in vivo. The interaction between breast cancer cells with bone stromal cells was studied in gelatin-methacrylate hydrogel environment with bone-mineral-mimetic nano-hydroxyapatite (nHA) composite^45^. The co-culture model showed enhanced proliferation of breast cancer cells and reduced proliferation of mesenchymal stromal cells (MSCs)/osteoblast cells. By changing different biophysical parameters (such as thickness, composition, etc.) of scaffolds, this study demonstrated the metastatic behavior of breast cancer cells in the presence of bone cells. Breast cancer cells, co-cultured with bone marrow stem cells in a 3D bioprinted hydrogel-hydroxyapatite composite scaffolds^46^, favored spheroid formation, and proliferation of breast cancer cells, signifying the influence of stem cells in metastatic progression of cancer cells. Moreover, cancer cells in 3D showed higher resistance to chemotherapy drug in comparison to cells in 2D (ref. ^47^). Such models can aid in predicting the role of metastatic behavior of cancer cells on remodeling of bone tissue.

### Pancreatic adenocarcinoma

Pancreatic adenocarcinoma is an exocrine tumor occurring in the cells that line up the pancreatic duct. It spreads rapidly, has poor prognosis, and is undetected in early stages of the disease. This combined with therapeutic resistance makes the treatment of the disease extremely difficult. As 2D models do not recapitulate the pancreatic microenvironment correctly, a 3D bioprinted organotypic pancreatic adenocarcinoma model was fabricated using pancreatic cancer cells inside a microenvironment, consisting of endothelial and pancreatic stellate cells by Sears et al.^48^. In another study by Langer et al., efforts were also made in bioprinting scaffold-free tumors using patient-derived cells to examine if the growth and development of pancreatic cells could be recapitulated in vitro^43^. This model was able to mimic many features of tumor, including response to extrinsic signals and in vivo morphology.

### Ovarian and cervical cancer

**Fig. 4:**
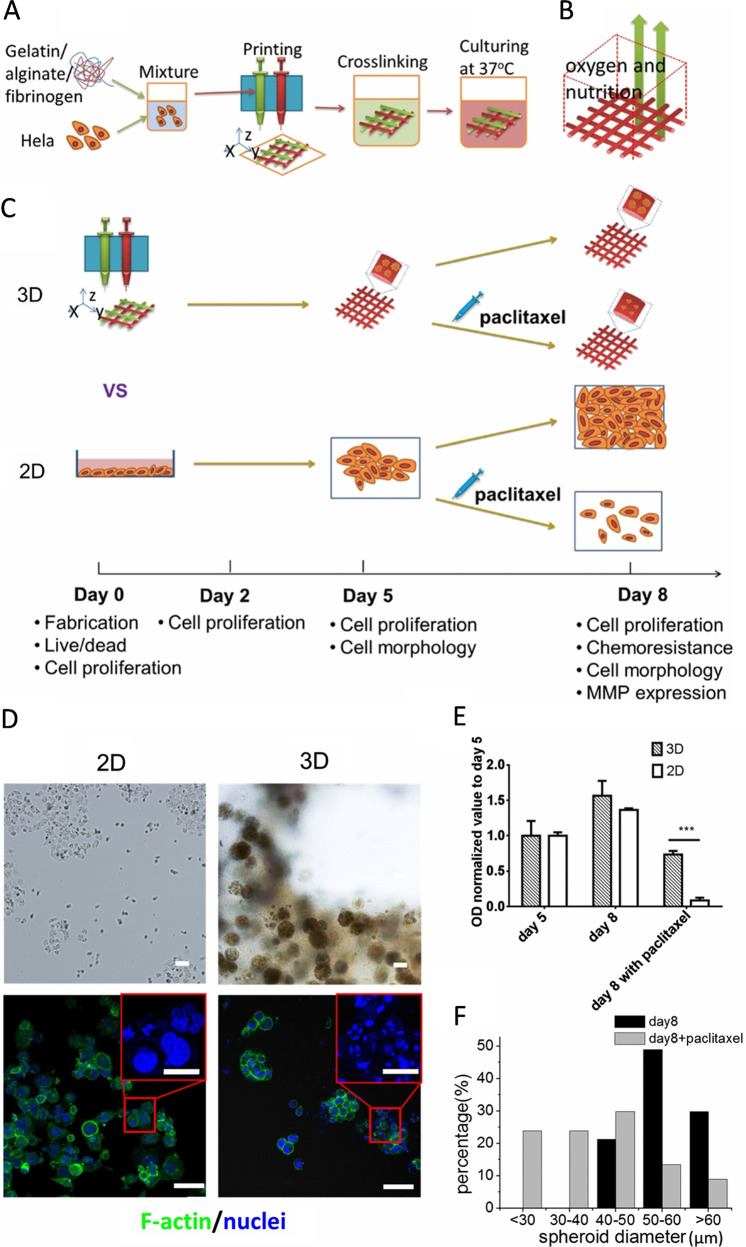
A 3D bioprinted cervical cancer model. **a** 3D Hela/hydrogel construct fabricated using EBB. **b** Schematic representation of 3D printed construct. **c** Schematic description of the timeline followed for the fabrication of tissue. 3D bioprinted constructs as well as 2D planar samples were all cultured for five days followed by paclitaxel addition and culture for the next three days. **d** Cell morphology after paclitaxel treatment on 3D bioprinted and 2D planar sample. **e** Cellular metabolic activity after paclitaxel treatment shows chemoresistance for 3D samples. **f** Comparison of spheroid diameters in the hydrogel with and without the addition of paclitaxel. ^***^p < 0.001 by *t*-test (scale bar, 50 µm; scale bar in enlarged images, 20 µm; reproduced/adapted with permission from ref. ^51^).

**Fig. 5:**
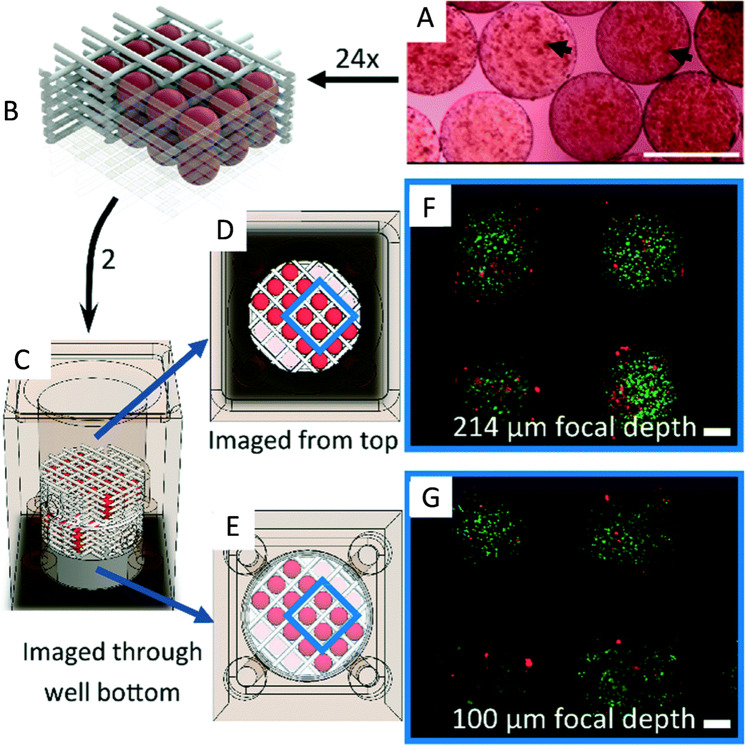
A 3D bioprinted ovarian cancer model. **a** GelMA μS loaded with cells. **b** µS are assembled in the polycaprolactone (PCL) scaffold. **c** Assembled PCL scaffold was carefully loaded into a bioreactor, imaged from **d** top and **e** bottom, and **f**, **g** stained with calcein-AM and propidium iodide in PBS (both at 1 μg mL^−1^) for live (green)–dead (red) staining (scale bars, 200 μm; reproduced/adapted with permission from ref. ^52^).

Ovarian or cervical cancer usually goes undetected in early stages and mostly diagnosed after the cancer spreads to other organs. Similar to other cancer types, ovarian or cervical cancer also presents a heterogenous microenvironment, which is difficult to recapitulate using 2D models. In this regard, a high-throughput bioprinting system was used to construct a tumor model on Matrigel substrate by co-bioprinting ovarian cancer cells (OVCAR-5) and fibroblasts (MRC-5). The authors observed the alteration in formation of micro-nodules by OVCAR-5 cells in the co-culture model as well as a parallel model, in which certain distance was maintained between OVCAR-5 and fibroblast cells^49^. These 3D models exhibited different sensitivity to co-therapy of benzorphyrin-mediated photodynamic therapy and carboplatin administration^50^. In another study, human cervical carcinoma (HeLa) cells, encapsulated in a mixture of fibrinogen, alginate, and gelatin, were bioprinted using EBB method, as shown in Fig. 4 (ref. ^51^). In comparison to 2D, differences were evident in the bioprinted model with respect to proliferation, matrix metalloproteinase (MMP), and chemoresistance to paclitaxel markers. 3D spheroid formulation was also observed, possibly due to the enhanced intercellular and cell–material interactions. Biofabricated ovarian cancer models (Fig. 5) have also been developed using a LBB method, which demonstrated the perfusion characteristics and metabolic behavior of ovarian cancer cells^52^. A dose–response curve was generated for DOX and application of in situ microscopy was also demonstrated for quantitative bioassay with the model. The application of the model was also demonstrated for imaging of microparticle flow inside hydrogel constructs.

### Hepatocarcinoma

**Fig. 6:**
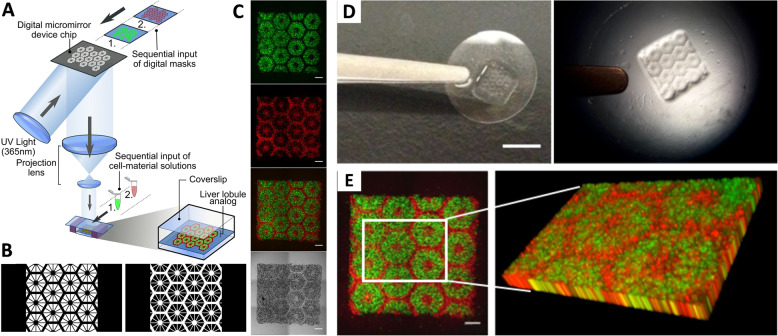
Bioprinting a triculture hepatic construct. **a** Schematic representation of a two-step 3D bioprinting approach, in which hiPSC-HPCs were patterned by the first digital mask followed by patterning using a second digital mask containing supporting cells. **b** Grayscale digital masks designed for two-step bioprinting. The white patterns represent the light reflecting patterns for photopolymerization. **c** Fluorescent and bright field Images (5×) of patterns made from fluorescently labeled hiPSC-HPCs (green) in 5% (wt/vol) GelMA, and supporting cells (red) in 2.5% (wt/vol) GelMA with 1% GMHA on day 0 (scale bars, 500 µm.) **d** A piece of coverslip containing 3D bioprinted hepatic construct (scale bar, 5 mm.) **e** 3D reconstruction of a bioprinted patterned construct (scale bar, 500 µm; reproduced/adapted with permission from ref. ^55^).

Hepatocellular carcinoma (HCC) is one of the most common malignant forms of cancer and the second largest cause of mortality worldwide^53^. It is usually characterized by the development of stiff hepatocellular nodules, where the liver ECM stiffness is found to be greater than that of healthy liver parenchyma^54^. A 3D hepatocarcinoma model was fabricated by Ma et al. through a two-step stereolithographic bioprinting method as illustrated in Fig. 6 (ref. ^55^). They patterned liver dECM with tailorable mechanical properties, which formed hexagonal lobular structure containing human iPSC-derived hepatic progenitor cells (HPCs) and endothelial cells. This served as a platform for HCC progression study. The iPSC-derived HPCs in 3D triculture exhibited differential expression of genes correlated with secretion of liver-specific proteins like transthyretin, hepatocyte nuclear factor 4α, albumin corresponding to the different stages of cellular maturation inside the constructs. The cells in 3D constructs also exhibited increased basal cytochrome P450 levels, indicating adult hepatocytes maturation and induction by rifampicin of certain subtypes of this enzyme system was also evidenced, suggesting development of a physiologically relevant construct. This model can be further improved to large-scale hepatic disease model by inclusion of ECM cues, patient-derived diseased cells, and functional vasculature. In another study, Kolesky et al.^56^ established a 3D printed hepatocarcinoma model using an alternative approach. They used a multi-nozzle printing device through which Pluronic F127 (a tri-block copolymers of poly(ethylene oxide)-poly(propylene oxide)-poly(ethylene oxide)) was printed using one nozzle head, and other nozzles were used for bioprinting HepG2 and human umbilical vein endothelial cell (HUVEC) containing hydrogels. Thus, a highly scalable platform was developed for generating engineered tissue constructs containing vascular channels and multiple cell types inside the ECM milieau.

Table 1 summarizes the cancer models fabricated with the use of different bioprinting techniques and bioink materials.

## Integration Of bioprinting with microfluidics

Bionic engineering integrating the principles of microfluidics and 3D bioprinting with co-culture techniques can improve the effectiveness of in vitro tumor models for disease biology and drug testing. For developing the complex heterogeneous tumor microenvironment, the integration of microfluidics with bioprinting is an emerging approach. Fluid flow can be introduced through microfluidic systems, which recapitulates the dynamic tumor microenvironment. Studies have proven that interstitial fluid flow around the tumor tissue creates shear stress, which arrests the cell cycle progression of cancer cells^24^ and cancer cells also exhibit tendencies to migrate toward the direction of fluid flow^57^. Microfluidic system can create different flow patterns and chemical gradients, which is effective to study the metastatic behavior of cancer cells.

Huang et al. fabricated microchannels in dimensions of 25, 45, and 120 µm using bioprinting, and studied the migration of cancerous HeLa cells^15^ and noncancerous 10T1/2 cells. The migration speed of HeLa cells was increased in smaller channels, whereas no differences in the speed was observed for 10T1/2 cells. In another work, Soman et al. fabricated polyethyleneglycol diacrylate (PEGDA)-based 3D log-pile structures^14^ and tuned the stiffness by changing the prepolymer concentration. Transformed cells showed higher migration velocity in this matrix as compared to normal epithelial cells, a behavior which cannot be observed under 2D conditions^58^. The feasibility of microfluidics integration was exemplified by fabrication of a hepatoma model in which the cluster size could be controlled and demonstrated for efficacy testing of Metuzumab, an anti-CD147 monoclonal antibody. Compared to a bioprinted-only in vitro model, hepatoma cells in the dynamic microfluidic model proliferated at a faster rate. Simultaneously, compared to 2D models, 3D model exhibited higher drug dosage dependant tolerance of growth and metastasis, which bear closer resemblance to trends observed in preclinical animal experiments as well as clinical trials of anti-CD147 antibody drugs. Further, investigations revealed that if the said model was improved by including peripheral blood mononuclear cells (PBMC) as effector cells and antibody-dependent cell-mediated cytotoxicity in pharmacology of these antibody drugs, a higher effectiveness of the antibody-dependent cell-mediated cytotoxicity tests under same drug dose in the integrated microfluidic-3D bioprinting models could be obtained^59^.

The integration of bioprinting with microfluidics can be performed in two principal approaches. In the first approach, bioprinting can be performed with microfluidic-modified printing nozzles. This can overcome some of the limitations of conventional bioprinting methods like the ability to precisely deposit multiple cell types inside a single bulk tissue, and reduce the mechanical- or thermal-forces induced damages to cell phenotypes. Such microfluidic-modified bioprinting has been demonstrated for fabrication of vascular channels and aligned muscle tissue constructs^60^. In the second approach, bioprinting can be carried out directly on a microfluidic-patterned receiving well plates. Such models have been built for co-patterned hepatoma and glioma cells, in which the anticancer effect of a drug tegafur was analyzed on glioma cells after metabolized by HepG2 cells^61^. Bioprinting has also been employed for developing microfluidic chips. For example, direct cell writing has been used to fabricate micro-organs inside soft-lithography-generated polydimethyl siloxane (PDMS) microfluidic chips^62,63^. Direct cell writing is one of the most convenient methods to fabricate 3D tissues in conjuction with microfluidics. In a two-step process, direct cell writing first creates the 3D tissue and then incorporates a PDMS device pre-fabricated by soft-lithography^64^. Current in vitro models are insufficient for drug screening since the perfusion and microcirculation methods are inadequate to mimic the true biological scenario. Recently, ‘tumor-on-a-chip’ based devices have been coupled with bioprinted vasculatures and lymph nodes^65^. This device was a bioprinted blood and a lymphatic vessel pair (TOC-BBL) model, wherein blood vessels were printed as hollow perfusable tubes with both ends open whereas lymph nodes were fabricated as tubes with one side sealed or blinded. This was achieved by multilayered coaxial, concentric nozzle assembly and adjustments of the flow rates of the crosslinker and bioink solutions. The permeability of the vessels were tuned by selecting optimum bioink compositions. This system was used to simulate the complex transport mechanism used for drug screening in cancer microenvironment.

**Fig. 7:**
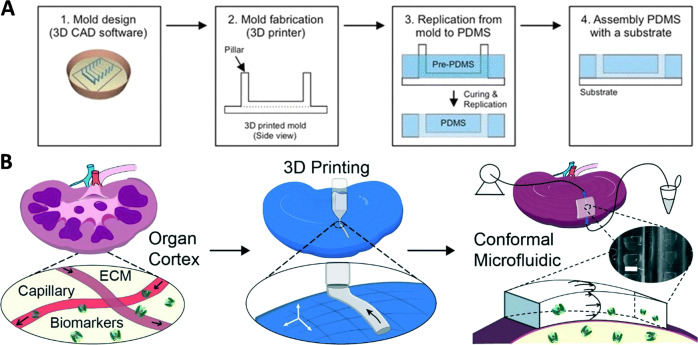
Integration of microfluidic devices with 3D bioprinted organ-on-a-chip models. (**a**) 3D printing of PDMS-based microfluidic chip (reproduced/adapted with permission from ref. ^66^). (**b**) Schematic representation of the microfluidic biopsy application of 3D printed conformal chips for isolating biomarkers from the organ cortex (scale bar, 500 μm) (reproduced/adapted with permission from ref. ^67^).

On the corollary, several microfluidic devices can themselves be fabricated by the use of 3D printing, as exemplified in Fig. 7a, b (refs. ^66,67^). Such methodology can be applied to deposit cells with multi-nozzle deposition systems over channels built by a digital micro-mirror technique^68^. Integration of multi-head bioprinting with a maskless solid-freeform fabrication system was also developed, which speeded up the fabrication process and eliminated toxic chemicals. Each 3D print-head can be used to deposit either of a photoresist for the circuit architecture; crosslinker for the photopolymer, helium and oxygen plasma deposition; and finally the bioink deposition inside microchannels^69^. 3D bioprinting has also been combined with low-temperature molding using A549/95-D cell and gelatin-sodium alginate based bioink to develop a model for exploring lung cancer invasion, where bioprinted cells showed increased migration potential compared to cells in 2D as determined from histochemical, scratch test and genetic assays^70^.

In precision oncology, tumor-on-a-chip models can overcome the absence of complexity of in vitro models and hence improve the prediction of drug screening, systemic drug toxicity and whole-organ drug pharmacology^71^. While organoid or tumoroid models rely on self-organization, organ-on-chip models can better recapitulate tumor microenvironment by providing greater control over spatial confinement and multi-organ interconnections. Such models with built-in vasculature can be fabricated enabling 3D parenchymal organization surrounded by endothelial cell-coated perfusable microchannels, which can be used for studying perfusability and cancer metastasis in multiple organs. Skardal et al. have demonstrated a microfluidic device for individualized therapy by developing a gut cancer metastasis model containing co-culture of malignant colon cells with epithelial cells on a thiolated hyaluronic acid, thiolated gelatin, and PEGDA-based hydrogel. The epithelial cells and transformed colon cancer cells were used to mimic a primary tumor foci site. The construct was then connected to a liver HepG2 culture, by a circulating fluid flow, to act as the secondary metastasis site downstream^72^. The mechanical stiffness of these hydrogels could be varied by changing their degrees of polymerization, which was used to demonstrate that metastasis potential of the cells increased as the stiffness of the hydrogel was decreased^72^.

## Future outlook

### 3D bioprinting of biomimetic microenvironment

Among different fabrication techniques to recapitulate the architecture and microenvironment of tumor tissues, 3D bioprinting is advancing at a greater pace^73,74^. However, challenges still remain in terms of choice of suitable bioink, bioprinting time, dimension of bioprinted tissues^75^, etc. to render truly cancer mimetic models suitable for industrial applications. From aforementioned examples and studies conducted on different tumor models, it is evident that selecting a suitable bioink is one of the most crucial prerequisites for mimicking the tumor microenvironment. Absence of a universal bioink, and hence the use of customized bioinks and difference in cell seeding densities make the correlation of the outcomes difficult^76^. dECM (refs. ^77,78^) or spheroids have been recently used as bioinks for developing in vitro models and maintaining the tumor microenvironment^79^. Such dECM plays an important role in revealing cell–ECM and cell–cell interactions, as well as genetic mutation in culture. The stimuli-sensitive hydrogel-based bioinks are also used to mimic the dynamic changes of tumor microenvironment^80^. However, several recommendations have already emerged in the literature. For example, in bioprinting cancer models including co-culture, scaffold-free bioprinting approach is more suitable, and hence DBB with low viscosity should be the preferred method. DBB is also more suitable to create temporo-spatial gradients in drug administration. DBB with low viscosity bioinks is also advantageous for HTS since issues of nozzle clogging leading to batch-to-batch inhomogeneity may be reduced by this method^81^. 3D organotypic models comprising the vascular structures are one of the most realistic models for studying cancer metastasis and anticancer drug screening. In addition, the other main challenge in the development of tumor microenvironment is the use of immortalized cell lines, which get manipulated in long-term culture conditions as they undergo numerous passages. On the other hand, patient-derived primary cells conserve the heterotypic environment of native tumor niche and are more useful for developing personalized therapeutics^43^. Though, the ideal bioink for patient-derived cells are yet to be standardized with respect to efficient mixing of the components and matching of the mechanical properties.

### Bioprinted cancer models as a preclinical screening tool for immunotherapy

The tumor microenvironment consists of cancer cells, stromal tissue, and various immune cells, such as T cells and macrophages, the composition of which may also vary from patient to patient. The interactions between tumor and immune cells in this microenvironment is thought to play a critical role in the cancer development, progression, and control^82^. In recent years, strategies harnessing tumor-infiltrating T cells have become a remarkably effective treatment option for a subset of patients with cancer. In addition, cancer cells often create an immunosuppressive microenvironment, which can inhibit the effector functions of antitumor immune cells. However, many key features of interactions between cancer and immune cells, and how these interactions affect tumor growth, are poorly understood. Indeed, our limited understanding of the complex interplay between the tumor microenvironment and host immune response is a major barrier to improving immunotherapeutic strategies, and defining predictive biomarkers for clinical benefit.

Appropriate balance between suppressive versus cytotoxic immune responses within the tumor microenvironment correlate with a good prognosis for cancer patients^83,84^. Immunocompromised microenvironments that suppress immune responses benefit cancer, whereas the presence of proinflammatory and cytotoxic T cells result in better clinical outcomes^84^. While recruitment of CD8+ cytotoxic T cells and inflammatory IFN+ CD4+ (Th1) subsets into the tumor microenvironment correlates with better prognosis, high infiltration of regulatory T cells are associated with unfavorable prognosis in about half of the cases, whereas in the other half, they may be associated with good prognosis or show no impact^84^. Myeloid lineage cells, such as macrophages or dendritic cells (DCs), are also found abundantly in the tumor microenvironment. Although their role is not fully clear, they impact recruitment of other cells, remodeling the tissue, and activating or suppressing antitumor T cell responses^85^.

Patients with several cancer types, such as melanoma, non-small cell lung cancer, and renal cell carcinoma, have greatly benefited from checkpoint inhibitor therapy, such as anti-PD1 or anti-CTLA-4 (refs. ^86–89^). However, these treatments are only effective in a subset of patients and other cases, such as breast cancer, the benefits of immunotherapy are currently under investigation and not yet clear^90^. These clinical results highlight the potential and gaps in our understanding of the molecular and cellular nature of the tumor–immune interactions within the tumor microenvironment. Thus, understanding the balance of immune responses and how inhibitory or stimulatory receptors modulate T cell activation in a tumor microenvironment could be critical to improving cancer immunotherapy. Further, in the different tissues in cancer, T cells and other innate cell types can exhibit marked differences in phenotype and functions due to cell-to-cell interactions. Indeed, variations in expression of CD161(+) nonclassical T cells, CD4+ and CD8+ cells have been identified in lymph nodes, spleen, the mammary lymph nodes, and the tumor mass^91^. To model the spatial compartmentalization and migration behavior of immune cell leading to the development of metastasis niches in cancer, microfluidic platforms have already proved beneficial and their integration with bioprinting is foreseen^92–95^. In this regard, bioprinting could be employed to establish a gradient of antigen-presenting cancer cells encapsulated in a hydrogel. Further, this could potentially be combined either with microfluidic channels or with channels printed using sacrificial inks, to establish a dynamic environment within the hydrogel constructs^94,96^. Immune cells such as PBMC (ref. ^97^), DCs, macrophages, cytotoxic CD8+ T cells could be perfused through the fabricated channels to study migration of these cells toward cytokine producing cancer cells^93,94^. These immune cells have already shown to play a major role in cancer prognosis^98^. Stromal cells such as cancer-associated fibroblasts could also be bioprinted with the cancer cells to understand their indispensable role in regulating immune response via the secretion of cytokines^99^. However there are several challenges in incorporating dynamic conditions through mechanically weak hydrogels. A major limitation is maintaining channel integrity over long dynamic culture periods as cancer cells and stromal cells secrete MMPs, which gradually degrade the hydrogel microenvironment. Thus, long-term viability and activity of immune cells under such conditions would also be affected. Furthermore, bioprinting the entire model would require combination of several different bioprinting modalities, such as droplet, extrusion, etc. into one system, which eventually increases the complexity of the printing. Thus, manufacturability as well as scalability needs to be overcome for proper integration of bioprinting and immunotherapy.
